# Femur Fracture in a Newborn During Emergency Cesarean Delivery: A Case Report

**DOI:** 10.1002/ccr3.70084

**Published:** 2025-01-06

**Authors:** Yeshey Dorjey, Abhisek Pradhan, Gitanjali Lamichaney

**Affiliations:** ^1^ Department of Obstetrics and Gynaecology Phuntsholing General Hospital Chukha Bhutan; ^2^ Department of Orthopedic Surgery Phuntsholing General Hospital Chukha Bhutan; ^3^ Department of Pediatrics Phuntsholing General Hospital Chukha Bhutan

**Keywords:** birth injury, breech presentation, cesarean section, femoral fractures, newborn

## Abstract

Birth injury occurs when the delivery process is not appropriately attended, and the use of improper techniques or maneuvers while conducting the delivery. Cesarean delivery is considered safe as compared to vaginal for the breech presentation. However, this case reports a case of femur fracture of a newborn that occurred during an emergency cesarean section performed for breech presentation. This highlights cesarean section for malpresentation is not completely safe.

**Summary**

- A femur fracture occurred in a newborn delivered via emergency cesarean section for breech presentation.
- This reminds the attending obstetricians or the birth attendants to assist the delivery process appropriately using correct maneuvers with adequate force.
- This case also highlights that cesarean delivery is not a safe alternate route of delivery in the malpresentation of the fetus.

## 1 Introduction

Birth injury is defined as the structural destruction or functional deterioration of the newborn's body secondary to mechanical force during labor, delivery, or both [1]. In general, the incidence of birth injury is 3–11 per 1000 live births [1, 2].

There are several risk factors for birth injury, including fetal factors, maternal factors, and delivery mechanisms. Fetal factors include macrosomia, abnormal presentation, and prematurity; maternal factors include extreme maternal age, multiple pregnancies, primigravida, and distorted pelvic anatomy; and the use of inappropriate forceps and vacuum for delivery increases the risk of birth injury [3].

Birth injury can occur on any part of the body of the newborn and can occur during both vaginal and cesarean delivery. A birth injury can be mild such as abrasion on the skin, or severe such as intracranial hemorrhage. The most common birth injury reported during vaginal delivery is cephalohaematoma [2], and skin lacerations are the most common injuries reported following cesarean delivery [4]. Other injuries reported were caput succedaneum, clavicular fracture, Erb's palsy, humeral fracture, shoulder dislocation, and facial laceration [2].

Fractures of the femur in newborns are uncommon and are reported to occur in 0.1 per 1000 live births [5]. There are few cases of femur fracture in newborns during vaginal breech delivery reported in the literature [6], and one case of bilateral femur fracture is reported in a fetus with a malformation complex with stiffness [7]. The mechanism of femur fracture during vaginal breech delivery is well described, as it usually occurs because of difficult breech delivery [8]. Cesarean delivery is presumed to be safer in delivering a fetus in malpresentation and reduces the risk of long bone fractures. This work reports a case of femur fracture in a newborn delivered by emergency cesarean section.

## 2 Case History/Examination

A 32‐year‐old woman, G2P1, at 38^+1^ weeks with breech presentation was admitted to the maternity ward in labor pain. This was a planned pregnancy. A dating scan was performed at 11^+2^ weeks of gestation, and an anomaly scan was conducted at 19^+4^ weeks which revealed a normal fetus with normal extremities with visualization of four long bones corresponding to the gestation period. She received folic acid, iron, vitamin, and calcium supplementation during antenatal period. She had no other medical co‐morbidities, and her antenatal check‐ups were uneventful. A third‐trimester growth scan was performed at 36^+3^ weeks of gestation, and an estimated fetal weight (EFW) of 2950 g, anterior placenta, with an amniotic fluid index (AFI) of 8 cm in the breech presentation was found.

At 38^+1^ weeks of gestation, she was admitted to the maternity ward with reduced fetal movement and lower abdominal pain for 6 h duration. On examination, all the vital signs were within the normal range, and on obstetric examination, the symphysis fundal height was 32 cm, fetal parts were easily felt, longitudinal lie, and in breech presentation, the fetal heart rate was 160 b/min. Pelvic examination revealed that the cervical os was 4 cm dilated, uneffaced, posterior cervix, station—1, and the membrane was not felt, and a meconium‐stained liquor was noted.

## 3 Methods

An ultrasound scan was performed and revealed an EFW of 3150 g, anterior placenta grade III, an AFI of 4 cm, and the fetus in breech presentation. The fetal Doppler study showed a slightly raised umbilical artery pulsatility index (PI) of 1.03 and a normal middle cerebral artery PI.

**FIGURE 1:**
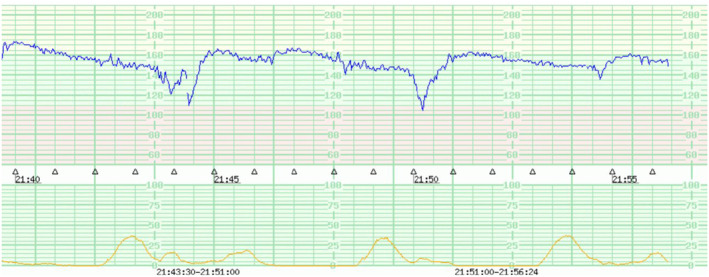
Admission i‐CTG showing late deceleration.

Hematological work‐up and other clinical examination findings were unremarkable. Admission i‐cardiotocography (i‐CTG) was performed and revealed reduced variability with late deceleration and uterine contraction (Figure 1).

A diagnosis of G2P1 at 38^+1^ weeks gestation in breech presentation, with labor pain with fetal distress was made.

**FIGURE 2:**
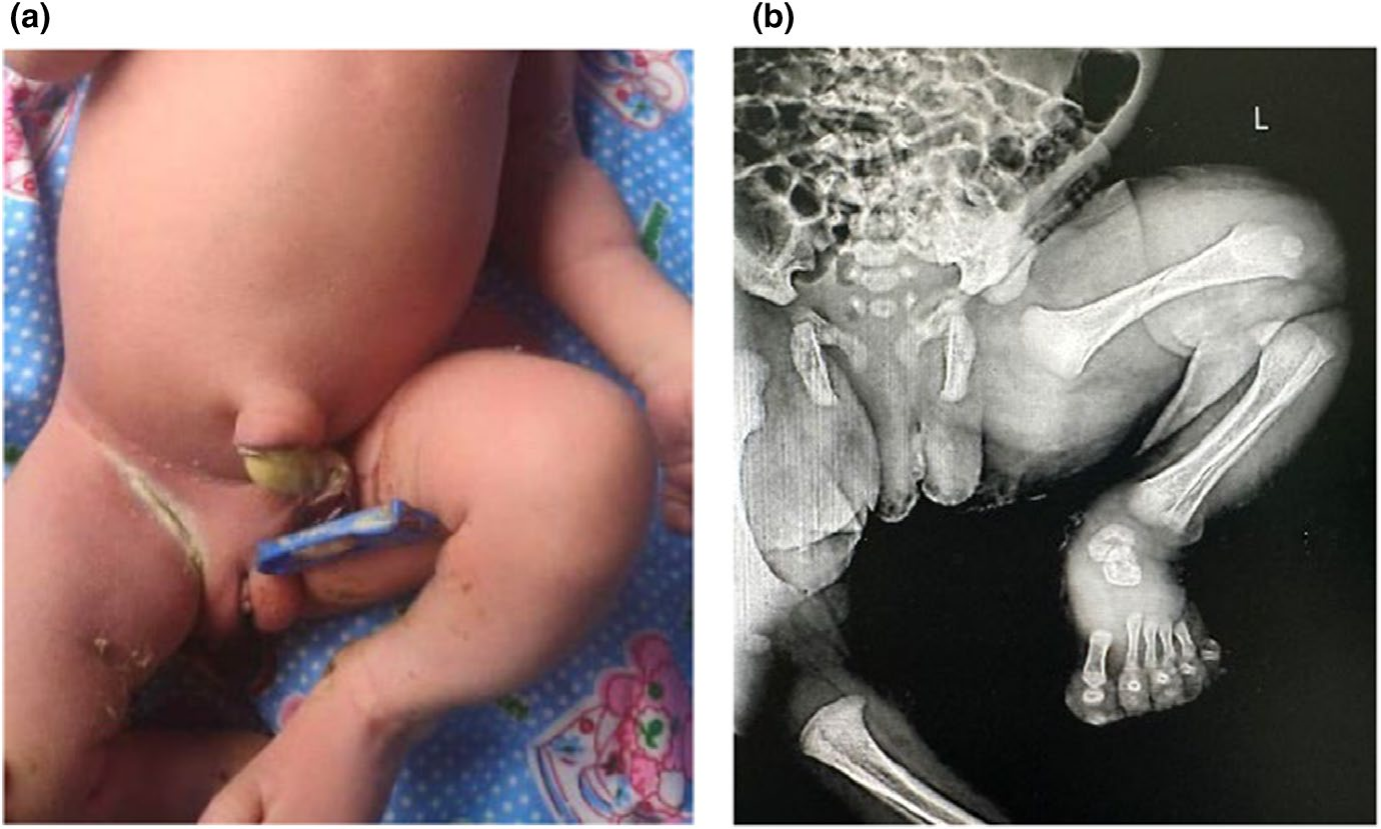
(a) Photo of a newborn showing the left leg in the flexion position. (b) A nondisplaced fracture of the shaft of the left femur on an X‐ray of the pelvis with the left hip.

Informed written consent was obtained from the patient, and the patient underwent an emergency cesarean section under spinal anesthesia. *Per‐operatively, scanty meconium‐stained liquor was present, and an extended or frank breech of both legs tightly struck inside the uterine cavity was noted*. A standard breech delivery by cesarean section was performed with slight difficulty in taking out the limbs. A live female baby weighing 3100 g was delivered with an Apgar score of 9 at 1 min and 10 at 5 min. At birth, no gross anomalies were detected on examination. After delivery, the baby was irritable with excessive crying and refused to breastfeed. On routine examination of the newborn on postnatal day 1, the baby was found to have swelling and reduced movement of the left leg, with the knee and hip in flexed position compared with the right leg. X‐ray of the pelvis with the left hip was performed, revealing an oblique nondisplaced fracture of the shaft of the left femur (Figure 2,b).

**FIGURE 3:**
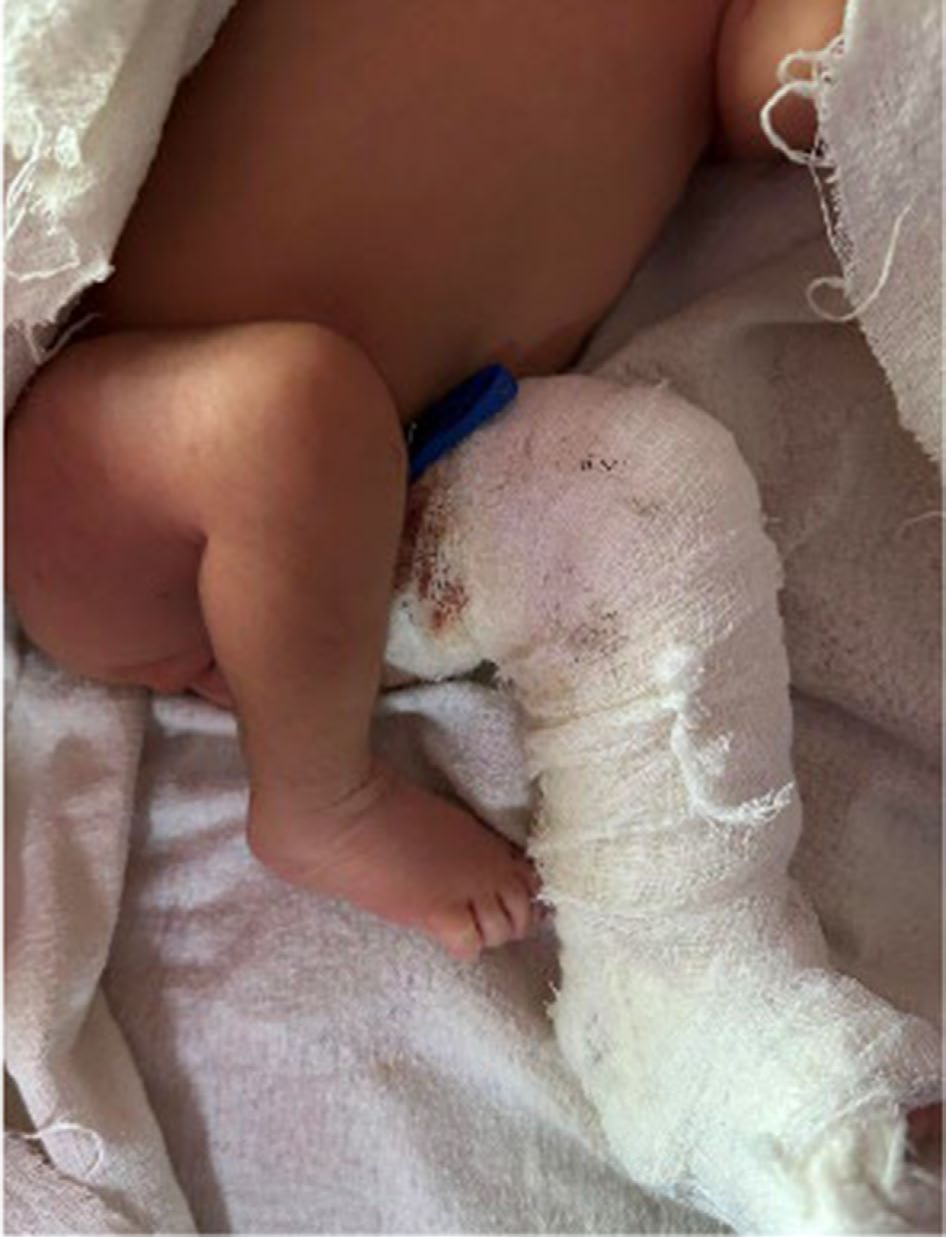
Photo showing a newborn with a posterior long‐leg splint applied to the left leg.

The orthopedic surgeon was consulted, and a posterior long‐leg splint was initially applied and later changed to a thermoplastic splint to immobilize the fractured limb (Figure 3).

On postnatal day 2, the baby developed neonatal jaundice with a transcutaneous bilirubin (TCB) level of 16 mg/dL and was given double surface phototherapy for 24 h. The mother was clinically stable and discharged on postoperative day 3. The baby was kept under close observation and discharged home on postnatal day 5 with a POP cast after establishing direct breastfeeding and resolving neonatal jaundice. The TCB level was 13 mg/dL at discharge.

## 4 Conclusion and Results

**FIGURE 4:**
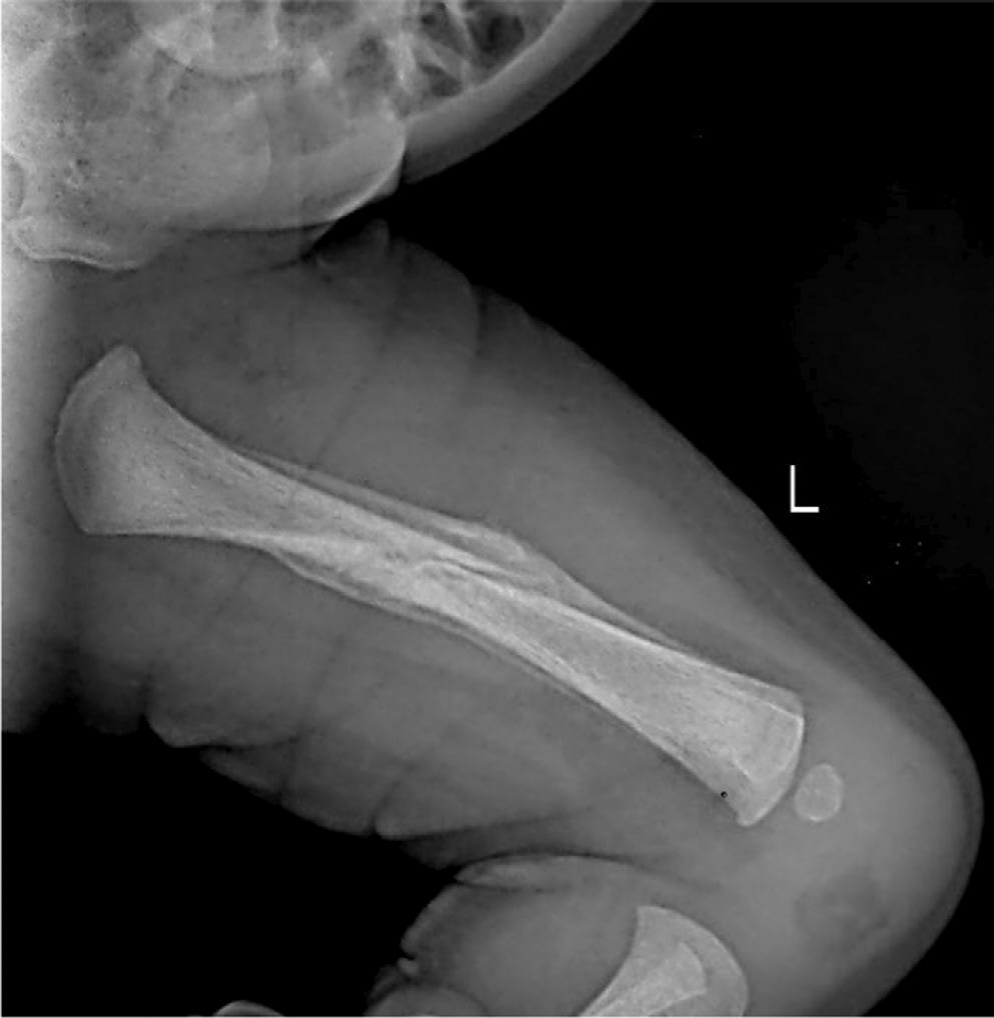
X‐ray of the left thigh taken after 2 weeks showing healing of the fractured femur with callus formation.

The baby was reviewed after 2 weeks at the pediatric and orthopedic unit and was found to be clinically stable. An X‐ray of the left femur was performed, and the results revealed healing of the fractured femur with a bridging callus formation (Figure 4). The thermoplastic splint was removed, and the baby was reviewed after 4 weeks and found clinically normal and the fracture healed completely.

## 5 Discussion

The current case highlights a femur fracture that occurred during the emergency cesarean delivery for the breech presentation. Birth injury or birth trauma can occur both during vaginal and cesarean delivery. A birth injury is usually reported following the delivery of malpresentation of the fetus, however, it can even occur when the fetus is in favorable (cephalic) presentation if the delivery process is not properly conducted or assisted. Even the skills and expertise of the attending obstetricians can contribute to the birth injury. For instance, birth injuries are usually reported following vacuum or forceps delivery [8]. Royal College of Obstetricians and Gynecologists (RCOG) guideline doesn't recommend a straightforward delivery route for breech presentation. Rather it stresses the importance of giving options of vaginal breech delivery versus elective cesarean delivery, and to opt for the most appropriate and safer route of delivery [9].

The most common birth injury reported is skin laceration during cesarean delivery and cephalohematoma following vaginal delivery. Fractures of the long bones of newborns often occur. Few cases of femur fractures have been reported in the literature following vaginal breech delivery, which is related to the use of improper maneuvers in the delivery of fetal legs [6].

In cases of malpresentation, compared with vaginal delivery, cesarean delivery is safe and recommended [10]. However, in the present case, a fracture of the femur occurred during cesarean delivery, which is otherwise considered a safer mode of delivery for breech presentation. During cesarean delivery, the fetus was found in an extended breech position tightly impacted inside the uterine cavity with scanty liquor. It was not easy to deliver the limbs of the fetus since there was no room to negotiate and perform the required maneuvers. This could be the possible reason for the fracture of the femur during delivery of the legs of the fetus. Clinically, all the points were in favor of fetal distress, and the baby was rapidly delivered. Rapid delivery of a fetus by cesarean section within 3 min from skin incision to delivery is another independent risk factor for fetal injury [4]. The long bones of the fetus are fragile and prone to fracture if inappropriate forces are applied or if incorrect maneuvers are used to deliver the extended breech. In addition, skeletal dysplasia with poor mineralization of bones could lead to easy fracture or present with fractures of bones during the intrauterine period [11]. However, in the present case, a detailed prenatal anatomical survey performed at 19 weeks of gestation revealed normal long bones in the fetus and excluded skeletal dysplasia.

Fractures or dislocations of the femur of a newborn sometimes go unnoticed at birth. Excessive crying or refusal to breastfeed and impaired movements of the affected limbs prompted the clinicians to perform further evaluations to diagnose the case [12]. On clinical examination, the fractured limb typically appears abnormally positioned or shorter in length than the unaffected leg. These presentations and clinical findings indicate a fracture or a dislocation of a long bone in the newborn. In the present case, the newborn had inconsolable crying and refused to breastfeed, and the left leg was found not moving and remained in a fixed position of flexion at the hip and knee joints. Later, an X‐ray of the pelvis and left femur confirmed the fracture of the left femur bone.

The management of birth trauma varies depending on the type of injury. Several nonoperative treatments are available for femur fractures in newborns, including a brace, Pavlik harness, Bryant skin traction, and a hip spica cast with or without traction. A few cases of femur fractures were managed with strapping applied to the thigh and legs [13]. The current case was managed with a posterior long‐leg splint initially followed by a thermoplastic splint applied over the thigh and leg. Treatment with a splint is not ideal in case of displaced neonatal displaced fracture, and it usually requires surgical correction [14]. The baby was reviewed after 2 weeks with an X‐ray report, which revealed a healing fracture with good bridging callus formation, which indicates that the fracture in babies heals faster without much complications.

Birth trauma is a preventable complication of delivery. Allowing a natural course of delivery without intervention is the best choice for the laboring mother, however, if the delivery course deviates from the expected course, birth attendant intervention is needed. Use of timely appropriate maneuvers, and handling gently while delivering a fetus in breech must be practiced by all the birth attendants to prevent long bone fractures during the delivery process.

## 6 Patient Perspective

Parents of the newborn were worried about the long‐term implications that might occur from femur fracture and said “What will happen to the baby in the future, and will it hamper baby growth?”

## Author Contributions

**Yeshey Dorjey:** conceptualization, data curation, methodology, supervision, validation, visualization, writing – original draft, writing – review and editing. **Abhisek Pradhan:** conceptualization, data curation, investigation, methodology, validation, visualization, writing – original draft, writing – review and editing. **Gitanjali Lamichaney:** conceptualization, data curation, formal analysis, validation, writing – original draft, writing – review and editing.

## Ethics Statement

The authors affirm that this manuscript adheres to the principles of medical ethics. All research involving human subjects was conducted with appropriate ethical approval, and informed consent was obtained from all participants. Any potential conflicts of interest have been disclosed.

## Consent

Written informed consent was obtained from the parents of the baby to publish this report in accordance with the journal's patient consent policy.

## Conflicts of Interest

The authors declare no conflicts of interest.

## Data Availability

Data is available on request from the corresponding author.
